# Stimulation or Cancellation of Ca^2+^ Influx by Bipolar Nanosecond Pulsed Electric Fields in Adrenal Chromaffin Cells Can Be Achieved by Tuning Pulse Waveform

**DOI:** 10.1038/s41598-019-47929-4

**Published:** 2019-08-08

**Authors:** Tarique R. Bagalkot, Normand Leblanc, Gale L. Craviso

**Affiliations:** Department of Pharmacology, University of Nevada, Reno School of Medicine, Reno, NV 89557 USA

**Keywords:** Membrane biophysics, Ion transport

## Abstract

Exposing adrenal chromaffin cells to single 150 to 400 ns electric pulses triggers a rise in intracellular Ca^2+^ ([Ca^2+^]_i_) that is due to Ca^2+^ influx through voltage-gated Ca^2+^ channels (VGCC) and plasma membrane electropores. Immediate delivery of a second pulse of the opposite polarity in which the duration and amplitude were the same as the first pulse (a symmetrical bipolar pulse) or greater than the first pulse (an asymmetrical bipolar pulse) had a stimulatory effect, evoking larger Ca^2+^ responses than the corresponding unipolar pulse. Progressively decreasing the amplitude of the opposite polarity pulse while also increasing its duration converted stimulation to attenuation, which reached a maximum of 43% when the positive phase was 150 ns at 3.1 kV/cm, and the negative phase was 800 ns at 0.2 kV/cm. When VGCCs were blocked, Ca^2+^ responses evoked by asymmetrical and even symmetrical bipolar pulses were significantly reduced relative to those evoked by the corresponding unipolar pulse under the same conditions, indicating that attenuation involved mainly the portion of Ca^2+^ influx attributable to membrane electropermeabilization. Thus, by tuning the shape of the bipolar pulse, Ca^2+^ entry into chromaffin cells through electropores could be attenuated while preserving Ca^2+^ influx through VGCCs.

## Introduction

Neuroendocrine adrenal chromaffin cells are neural crest-derived excitable cells involved in the sympathetic discharge of the catecholamines epinephrine and norepinephrine into the vascular system during the “fight or flight” response. *In vivo*, catecholamine secretion from chromaffin cells occurs when acetylcholine released from splanchnic nerve terminals stimulates nicotinic cholinergic receptors (nAChRs) that are ligand-gated Na^+^ channels. Na^+^ influx via nAChRs causes membrane depolarization leading to an elevation in intracellular Ca^2+^ concentration ([Ca^2+^]_i_) orchestrated by Ca^2+^ entry through several classes of voltage-gated Ca^2+^ channels (VGCCs; reviewed in García *et al*.)^1^. This sequence of events culminates in Ca^2+^-dependent exocytosis^2,3^.

As modified post-ganglionic sympathetic neurons that are easy to isolate and maintain in culture, isolated chromaffin cells have played a major role in characterizing mechanisms underlying stimulus-secretion coupling. Accordingly, our group has been using bovine adrenal chromaffin cells as a model system to explore the potential for high intensity, nanosecond pulsed electric fields (nsPEF) to serve as a new modality for neurostimulation^4^. Such short duration electric stimuli, particularly pulse durations that extend into the subnanosecond range, have the potential for remote, targeted delivery to tissues via antennas^5,6^, which could eliminate the need for invasive, contact electrodes. Moreover, the biophysical changes caused by nsPEF, one being the reversible formation of nanometer-diameter ion-conducting electropores (so-called “nanopores”) in the plasma membrane lipid bilayer^7–11^, may reveal novel mechanisms by which cell excitability can be altered. In adrenal chromaffin cells, as an example, we found that a single 5 ns, 5 MV/m pulse can stimulate these cells, producing an increase in [Ca^2+^]_i_ followed by Ca^2+^-dependent catecholamine release that is triggered by a mechanism that bypassed nicotinic receptors but still involved Na^+^ influx, membrane depolarization and activation of VGCCs^4,12–14^. Because tetrotoxin failed to block the response^4^ ruled out voltage-sensitive Na^+^ channels as the pathway of Na^+^ influx and suggested instead that Na^+^ was most likely entering chromaffin cells via plasma membrane nanopores, causing membrane depolarization^4^. A similar mechanism of electroporation-induced depolarization may also be involved in the stimulation of rat hippocampal neurons by 200 ns pulses^15^. Convincing evidence in support of this mechanism of cell excitability in chromaffin cells has been provided by whole-cell patch clamp recordings of nsPEF-evoked inward Na^+^ currents^16^, leading us to conclude that nanopores could be subserving a function typically attributed to voltage-gated Na^+^ channels by causing membrane depolarization to a level that was sufficient to activate VGCCs^4^. Notably, Ca^2+^ does not also enter chromaffin cells via this plasma membrane pathway^4,12,13^, indicating that like stimulus-secretion coupling mediated by acetylcholine, activation of VGCCs is solely responsible for Ca^2+^ influx. Of note also is that nsPEF-evoked cell activation occurs without evidence of the plasma membrane perturbations that promote uptake of non-permeant dyes such as YO-PRO-1^14^, release of Ca^2+^ from intracellular stores due to electroporation of membranes of Ca^2+^-storing organelles such the endoplasmic reticulum (ER)^4,12,13^, or adverse effects such as cell blebbing or cell swelling^14^. In addition to these findings from our group showing the potential for short duration nsPEF to be used for neurostimulation, Casciola *et al*. reported that 12 ns pulses applied to nerve fibers can elicit action potentials without causing nerve damage. Interestingly, the underlying mechanism of nsPEF-evoked excitability appears to be different in nerve than in chromaffin cells^17^.

While exploring other nsPEF parameters for stimulating chromaffin cells, a more recent study from our group revealed that exposing chromaffin cells to single longer duration nsPEF (≥150 ns) produced more robust and longer lasting Ca^2+^ responses also without evidence of cell swelling or cell blebbing, or uptake of YO-PRO-1^18^. However, the more pronounced rise in [Ca^2+^]_i_ evoked by these longer nsPEFs was found to be the result of Ca^2+^ entering the cells now by two pathways: (1) via VGCCs, which accounted for the bulk of Ca^2+^ influx (60–70%), and (2) Ca^2+^ influx through plasma membrane electropores^18^. Thus, the plasma membrane was no longer impermeable to Ca^2+^, coinciding with a similar finding of rapid influx of Ca^2+^ via nanopores in isolated rat hippocampal neurons exposed to 600 ns pulses^19^.

Recently, a new modality of nsPEF stimulation has been reported that enables fine-tuning of cellular responses that include nsPEF-evoked Ca^2+^ influx through plasma membrane nanopores. In this new nsPEF paradigm, the application of a second pulse of opposite polarity and equivalent duration delivered immediately after the first pulse (a symmetrical bipolar pulse) could attenuate or even abolish the response produced by the first pulse, a process termed bipolar cancellation. This phenomenon was first described by Pakhomov *et al*.^20^ in non-excitable Chinese Hamster Ovary (CHO) cells exposed to single or multiple unipolar or bipolar nsPEF^20^. In these cells, a 60 ns unipolar pulse triggered a rise in [Ca^2+^]_i_ that included nsPEF-induced Ca^2+^ influx from the outside the cell and Ca^2+^ efflux from the ER, attributed to a short-lived nanoelectroporation of the plasma membrane and ER membrane, respectively. At high stimulus intensities, Ca^2+^-induced calcium release (CICR) also contributed to the rise in [Ca^2+^]_i_^20^. When exposed to a symmetrical bipolar pulse (60 + 60 ns), CHO cells displayed much smaller Ca^2+^ transients than cells exposed to a 60 ns unipolar pulse, with each component that contributed to the Ca^2+^ response attenuated by the bipolar pulse. Bipolar cancellation was also demonstrated for longer duration pulses where a bipolar pulse (300 + 300 ns) was found to be less efficient for evoking increases in [Ca^2+^]_i_ compared with the corresponding unipolar pulse (300 ns).

In a subsequent report, Gianulis *et al*.^21^ evaluated the effects of a single or multiple nanosecond electric field unipolar and bipolar oscillation (referred to as NEFO) on membrane electropermeabilization and changes in membrane conductance in CHO and GH3 cells. Relative to unipolar NEFO, bipolar NEFO yielded lower rates of uptake of YO-PRO-1 and, as determined by whole-cell patch clamp electrophysiology, a reduced membrane conductance^21^. These results and those of others reporting a similar reduction of electropermeabilization by bipolar pulses were attributed to the ability of these pulses to cause less plasma membrane perturbations^20–24^. In light of these results, we considered the possibility that in chromaffin cells exposed to nsPEF ≥ 150 ns, Ca^2+^ influx due to plasma membrane electroporation might be attenuated/cancelled by applying a bipolar pulse. This could potentially mean eliminating an undesirable effect of longer duration nsPEF while preserving the ability of the cells to be activated in a manner similar to that evoked by a short duration nsPEF. The primary objective of the present study was to evaluate whether bipolar cancellation could be detected in chromaffin cells for nsPEF ≥ 150 ns by exploring the effect of symmetrical bipolar pulses (same duration and peak amplitude for each phase) and asymmetrical bipolar pulses (duration and/or peak amplitude of the negative phase different than the positive phase) on [Ca^2+^]_i_. Our results show that depending on the pulse waveform, bipolar pulses could either enhance or attenuate Ca^2+^ influx evoked by a 150 ns unipolar pulse. Moreover, attenuation of Ca^2+^ entry by bipolar nsPEFs appeared to involve the portion of Ca^2+^ influx attributable to membrane electropermeabilization and not Ca^2+^ influx via VGCCs. Thus, tuning the shape of the bipolar pulse attenuated undesirable Ca^2+^ entry into chromaffin cells through electropores while maintaining Ca^2+^ influx through VGCCs. These results have implications for the future development of nsPEF stimulation approaches for modulating cell excitability non-deleteriously since the reduced electroporation efficiency of bipolar compared with unipolar pulses expands the range of nsPEF parameters that can be used for this purpose.

## Results

### Symmetrical bipolar pulses stimulated Ca^2+^ influx to a greater degree than the corresponding unipolar pulse

**Figure 1:**
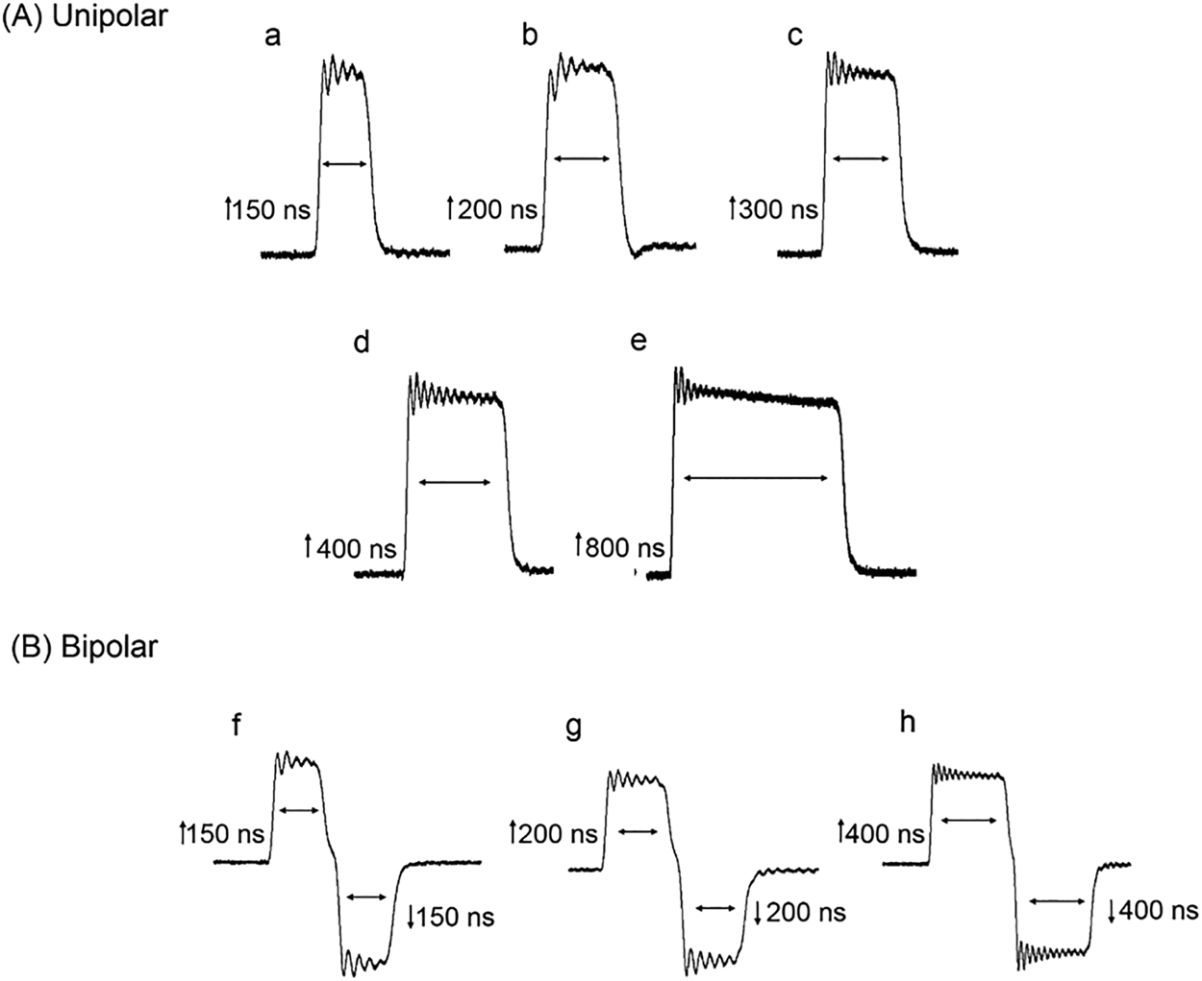
Oscilloscope traces of unipolar (**A**) and symmetrical bipolar (**B**) nsPEFs. (a–e) Unipolar nsPEFs were 150, 200, 400 and 800 ns in duration, all applied at an E-field of 3.1 kV/cm. (f–h) Symmetrical bipolar nsPEFs consisted of positive and negative phases of the pulse having the same duration, 150, 200 or 400 ns, and the same E-field of 3.1 kV/cm. Here and in all subsequent figures, the positive phase of the pulse was depicted by an upward arrow (↑) and the negative phase by a downward arrow (↓).

In non-excitable CHO cells, Ca^2+^ influx evoked by unipolar nsPEF could be “cancelled” by applying a second pulse of equivalent strength but opposite polarity and identical duration^11^. To determine whether bipolar cancellation of Ca^2+^ influx could be similarly achieved in excitable chromaffin cells, a systematic investigation of the impact of unipolar nsPEF on [Ca^2+^]_i_ was first undertaken followed by an analysis of the effects of the corresponding bipolar nsPEF on [Ca^2+^]_i_. Cells were exposed to a single unipolar pulse of 150, 200, 300, 400 or 800 ns duration (Fig. 1A) at an E-field amplitude of 3.1 kV/cm that we previously determined was just above threshold for evoking a Ca^2+^ response^18^. Single symmetrical bipolar pulses to which cells were exposed (Fig. 1B) were ↑150 + ↓150, ↑200 + ↓200 or ↑400 + ↓400 ns at an E-field amplitude of 3.1 kV/cm for each pulse polarity.

**Figure 2:**
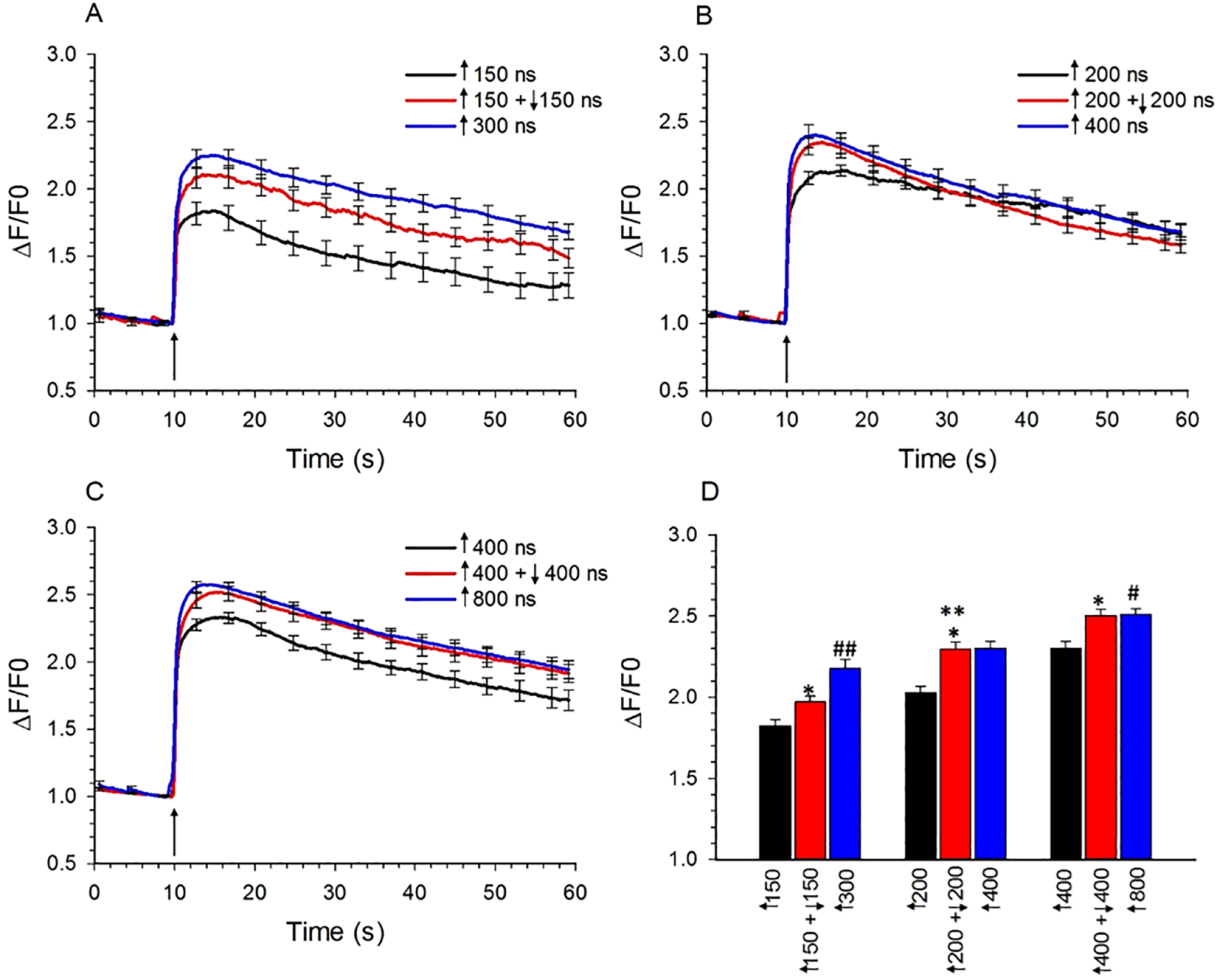
Comparison of the effect of unipolar pulses vs symmetrical bipolar pulses on [Ca^2+^]_i_. (**A**) Averaged cell responses ± s.e.m. (n = 17–60) for the rise in [Ca^2+^]_i_ in cells exposed to a unipolar pulse of 150 ns and 300 ns, and to a bipolar pulse with each phase lasting 150 ns. The E-field amplitude for each phase was 3.1 kV/cm and the arrow indicates when the pulse was delivered to the cells. (**B**) Same as in (**A**) except that cells were exposed to a unipolar pulse of 200 ns and 400 ns, and to a bipolar pulse with each phase lasting 200 ns (n = 30–35). (**C**) Same as in (**A**) except that cells were exposed to a unipolar pulse of 400 ns and 800 ns, and to a bipolar pulse with each phase lasting 400 ns (n = 21–35). (**D**) Bar graph showing the mean ± s.e.m. for the maximal increase in [Ca^2+^]_i_ for the responses shown in (**A**–**C**). ^*^p < 0.05, significantly different from the corresponding unipolar pulse; ^**^p < 0.01, significantly different from ↑150 + ↓150 and ↑400 + ↓400 bipolar pulse. ^#^*p* < 0.01, Significantly different from ↑150 + ↓150 and ↑200 + ↓200 bipolar pulse. ^##^*p* < 0.01, Significantly different from ↑150 + ↓150 bipolar pulse.

**Figure 3:**
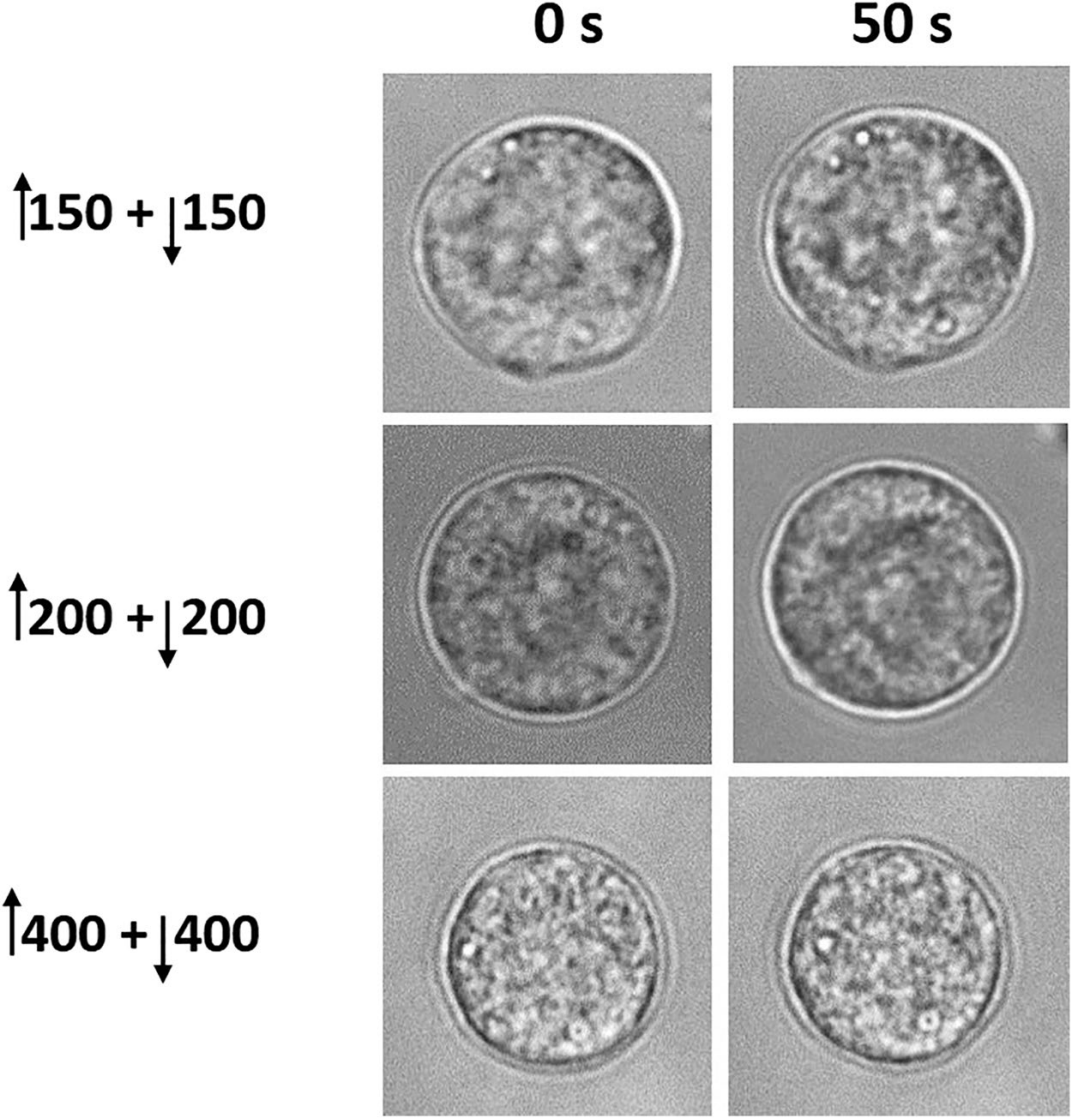
Representative bright field images of cells exposed to bipolar pulses. Images of the cells were captured before (0 s) and 50 s after exposure to a ↑150 + ↓150 ns, ↑200 + ↓200 ns, and ↑400 + ↓400 ns pulse. The E-field amplitude of each phase of each pulse duration was 3.1 kV/cm.

Figure 2A–C shows that the rise in [Ca^2+^]_i_ evoked by single unipolar pulses of 150, 200, 300, 400 or 800 ns duration occurred rapidly, increasing in amplitude with pulse duration and slowly declining to basal levels as reported previously^18^. Also shown is a comparison of the responses of the cells to unipolar vs bipolar pulses. Unexpectedly, bipolar pulses of ↑150 + ↓150, ↑200 + ↓200 and ↑400 + ↓400 ns evoked Ca^2+^ transients that were respectively larger by 18%, 26% and 15% than those elicited by the corresponding unipolar pulse (Table 1, Fig. 2D). In each case, the increase reached statistical significance (*p* < 0.01 for all pulse durations) and was not associated with adverse effects on cell morphology, such as cell swelling (Fig. 3). Thus, Ca^2+^ influx was not attenuated by symmetrical bipolar pulses, contrasting with previous findings reported by Pakhomov *et al*.^20^ in non-excitable CHO cells where Ca^2+^ influx triggered by unipolar pulses of 60 and 300 ns was cancelled by bipolar pulses of ↑60 + ↓60 and ↑300 + ↓300 ns, respectively.

**Table 1:** Summary of bipolar nsPEF parameters that evoked maximal stimulation or cancellation of Ca^2+^ influx elicited by a 150 ns unipolar pulse.

Condition	Effect on Ca^2+^ Fluorescence Intensity		
	Stimulation (%)	Cancellation (%)	*P* value
**1. Untreated cells**			
Symmetrical bipolar pulse^*^			
↑150 + ↓150 ns vs ↑150 ns	18	—	*<0.01*
↑200 + ↓200 ns vs ↑200 ns	26	—	*<0.01*
↑400 + ↓400 ns vs ↑400 ns	15	—	*<0.01*
Asymmetrical bipolar pulse^*^			
↑150 + ↓200 ns vs ↑150 ns	29	—	*NS*
↑150 + ↓400 ns vs ↑150 ns	37	—	*<0.05*
↑150 + ↓800 ns vs ↑150 ns	47	—	*<0.01*
Asymmetrical bipolar pulse^**^			
↑150 + ↓200 ns (2.4 kV/cm)	35	—	*NS*
↑150 + ↓200 ns (0.2 kV/cm)	—	4	*NS*
↑150 + ↓400 ns (2.4 kV/cm)	36	—	*<0.05*
↑150 + ↓400 ns (0.2 kV/cm)	—	27	*NS*
↑150 + ↓800 ns (2.4 kV/cm)	66	—	*<0.05*
↑150 + ↓800 ns (0.2 kV/cm)	—	43	*<0.05*
↑150 + ↓1000 ns (2.4 kV/cm)	86	—	*<0.05*
↑150 + ↓1000 ns (0.2 kV/cm)	—	36	*<0.05*
**2**. **VGCCs blocked**			
Symmetrical bipolar pulse^*^			
↑150 + ↓150 ns	—	60	*<0.05*
Asymmetrical bipolar pulse^**^			
↑150 + ↓1000 ns (3.1/0.4 kV/cm)	—	58	*<0.05*

^*^3.1 kV/cm each phase.

^**^3.1 kV/cm positive phase, varied amplitude and duration of the negative phase.

NS: Not significant.

**Figure 4:**
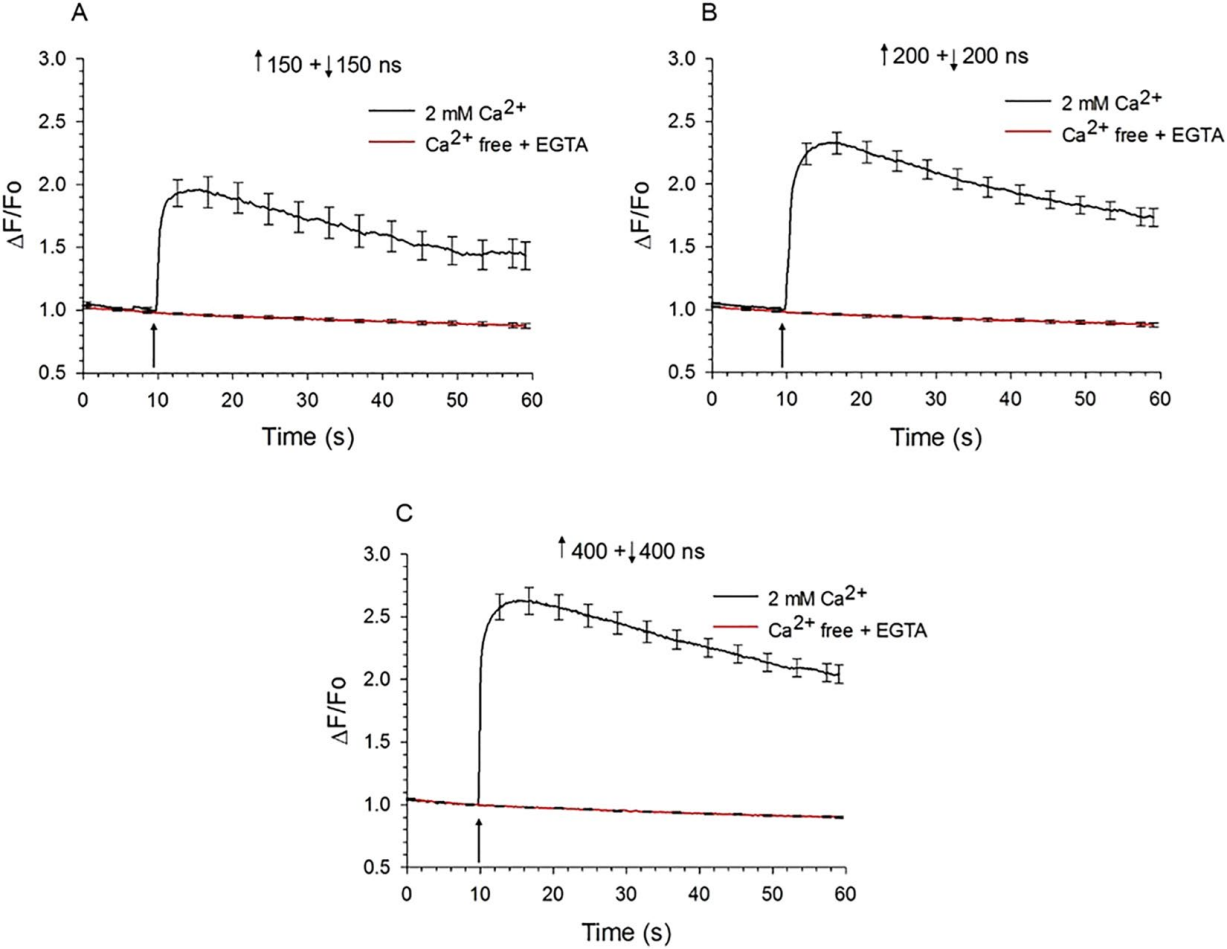
Effect of extracellular Ca^2+^ on Ca^2+^ responses elicited by symmetrical bipolar pulses. Averaged cell responses ± s.e.m for cells exposed to a (**A**) ↑150 + ↓150 ns pulse (n = 12–13), (**B**) ↑200 + ↓200 ns pulse (n = 10–14), and (**C**) ↑400 + ↓400 ns pulse (n = 6–11). Pulses were applied at an E-field of 3.1 kV/cm to cells in the absence (Ca^2+^-free BSS with 1 mM EGTA) or presence of 2 mM extracellular Ca^2+^. The arrow indicates when the pulse was delivered to the cells.

Since VGCC activation is an all-or-none cellular process, symmetrical bipolar pulses were most likely enhancing Ca^2+^ influx in chromaffin cells by causing greater membrane permeabilization rather than less, as reported for other cell types^20,21,25^. In other words, the negative pulse phase not only failed to cancel the electropermeabilizing effect of the first phase but also acted as a 2^nd^ electropermeabilizing stimulus. In support of this conclusion, we determined the effects of external Ca^2+^ removal on the Ca^2+^ response of chromaffin cells to this type of nsPEF. As shown in Fig. 4, bathing the cells in Ca^2+^-free BSS containing EGTA abolished Ca^2+^ transients elicited by ↑150 + 150 ns (Fig. 4A), ↑200 + ↓200 ns (Fig. 4B) or ↑400 + ↓400 ns (Fig. 4C) bipolar pulses with all pulse phases applied at an E-field of 3.1 kV/cm. These results indicate that regardless of pulse duration, the mechanism by which bipolar nsPEF exposure caused an increase in [Ca^2+^]_i_ in chromaffin cells was Ca^2+^ influx and not release of Ca^2+^ from internal stores caused by ER membrane electropermeabilization. While these results do not rule out the possibility that a portion of the Ca^2+^ transient evoked by nsPEFs was due to Ca^2+^ release from the ER via Ca^2+^-induced Ca^2+^ release (CICR), they unequivocally demonstrate an absolute requirement for a Ca^2+^ entry pathway to trigger the response.

The results of the experiments shown in Fig. 2 also demonstrate that Ca^2+^ responses evoked by bipolar pulses, like single unipolar pulses^18^, increased in amplitude with pulse duration. For example, Ca^2+^ responses evoked by ↑200 + ↓200 ns or ↑400 + ↓400 ns bipolar pulses were 55% or 33% larger, respectively, than those produced by ↑150 + ↓150 ns bipolar pulses (Fig. 2D) with the difference in magnitude reaching statistical significance (*p* < 0.01). Thus, the negative pulse phase acted as a 2^nd^ stimulus regardless of pulse duration.

When we compared Ca^2+^ influx induced by a bipolar pulse of ↑150 + ↓150 ns to a single unipolar pulse of 300 ns, which represents an equivalent amount of energy, the response elicited by the bipolar pulse was significantly smaller than that evoked by the unipolar pulse (21% reduction; *p* < 0.01). Thus, even though the negative phase of the ↑150 + ↓150 bipolar pulse acted as a 2^nd^ stimulus, Ca^2+^ influx was not comparable to that elicited by a single polarity 300 ns pulse. A possible explanation is that the positive phase of the bipolar pulse limited the extent to which the plasma membrane could be electropermeabilized by the negative phase of the pulse^22,25^. We also compared Ca^2+^ influx induced by a single bipolar pulse of ↑200 + ↓200 or ↑400 + ↓400 ns to a single unipolar pulse of 400 or 800 ns, which represents an equivalent amount of energy for each bipolar pulse, respectively. We found that the response triggered by a ↑200 + ↓200 ns vs a 400 ns pulse, or a ↑400 + ↓400 ns vs a 800 ns pulse was similar. Given the longer duration of these pulses relative to a ↑150 + ↓150 bipolar pulse or a unipolar 300 ns pulse, the Ca^2+^ indicator may have been saturated in each case, not allowing differences in [Ca^2+^]_i_ to be detected among the groups.

Taken as a whole, these results demonstrate that Ca^2+^ influx was not attenuated by symmetrical bipolar pulses. Instead, Ca^2+^ influx was enhanced. The next series of experiments investigated the effect of altering the shape of the bipolar pulse on Ca^2+^ influx by manipulating the parameters of the negative pulse phase.

### Increasing the duration of negative phase relative to the positive phase stimulated Ca^2+^ influx to a greater degree than a symmetrical bipolar pulse

**Figure 5:**
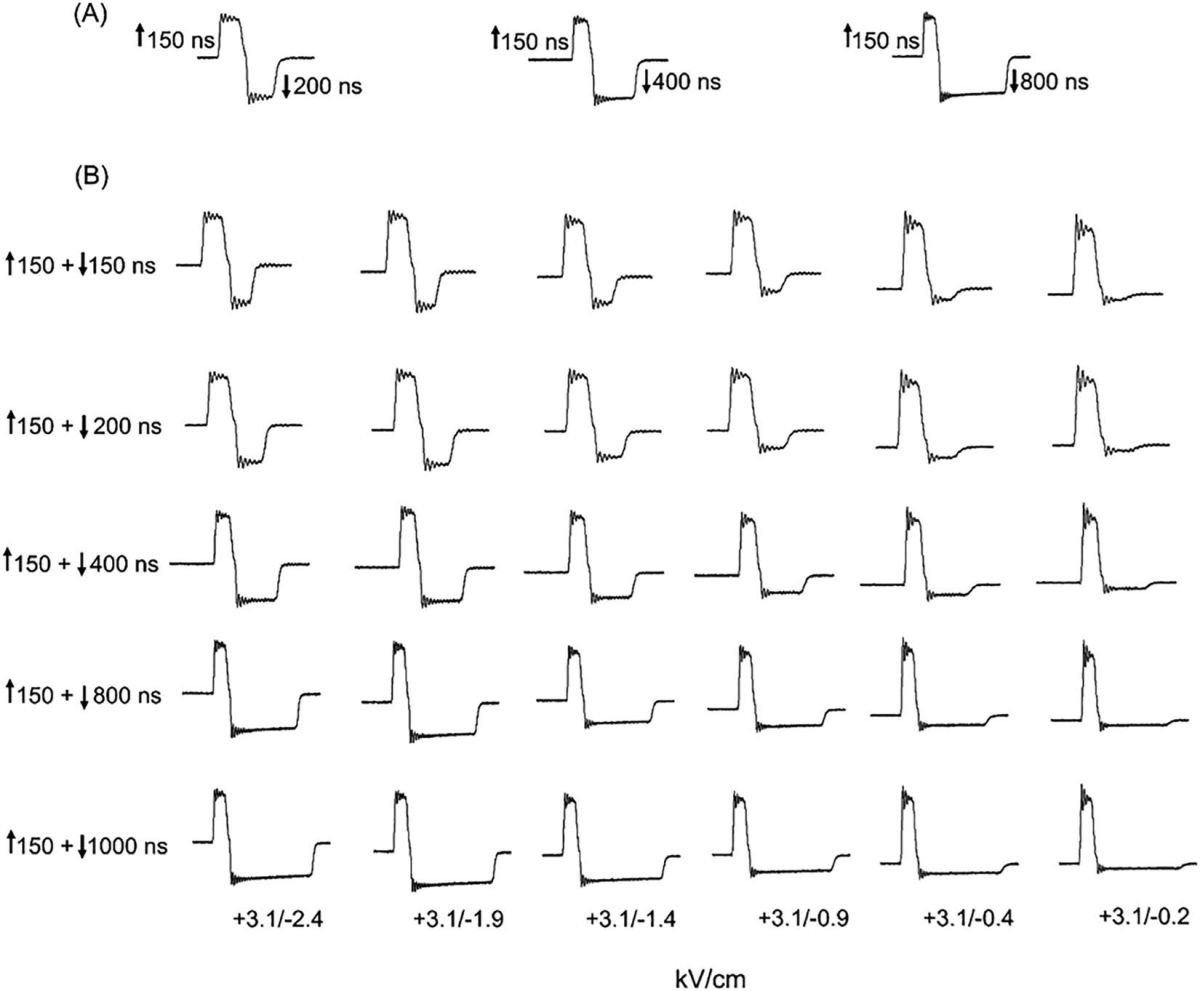
Representative oscilloscope traces of bipolar pulses having the same positive phase and negative phases but varying in duration and E-field magnitude. (**A**) Bipolar pulses in which the positive phase was 150 ns and negative phases were 200, 400 or 800 ns. In all cases, the E-field for each phase was set at 3.1 kV/cm. (**B**) Bipolar pulses in which the positive phase was 150 ns set at 3.1 kV/cm, and the negative pulse phase varied in amplitude from 2.4 kV/cm to 0.2 kV/cm (one E-field per column) and in duration from 150 to 1000 ns (one duration per row).

**Figure 6:**
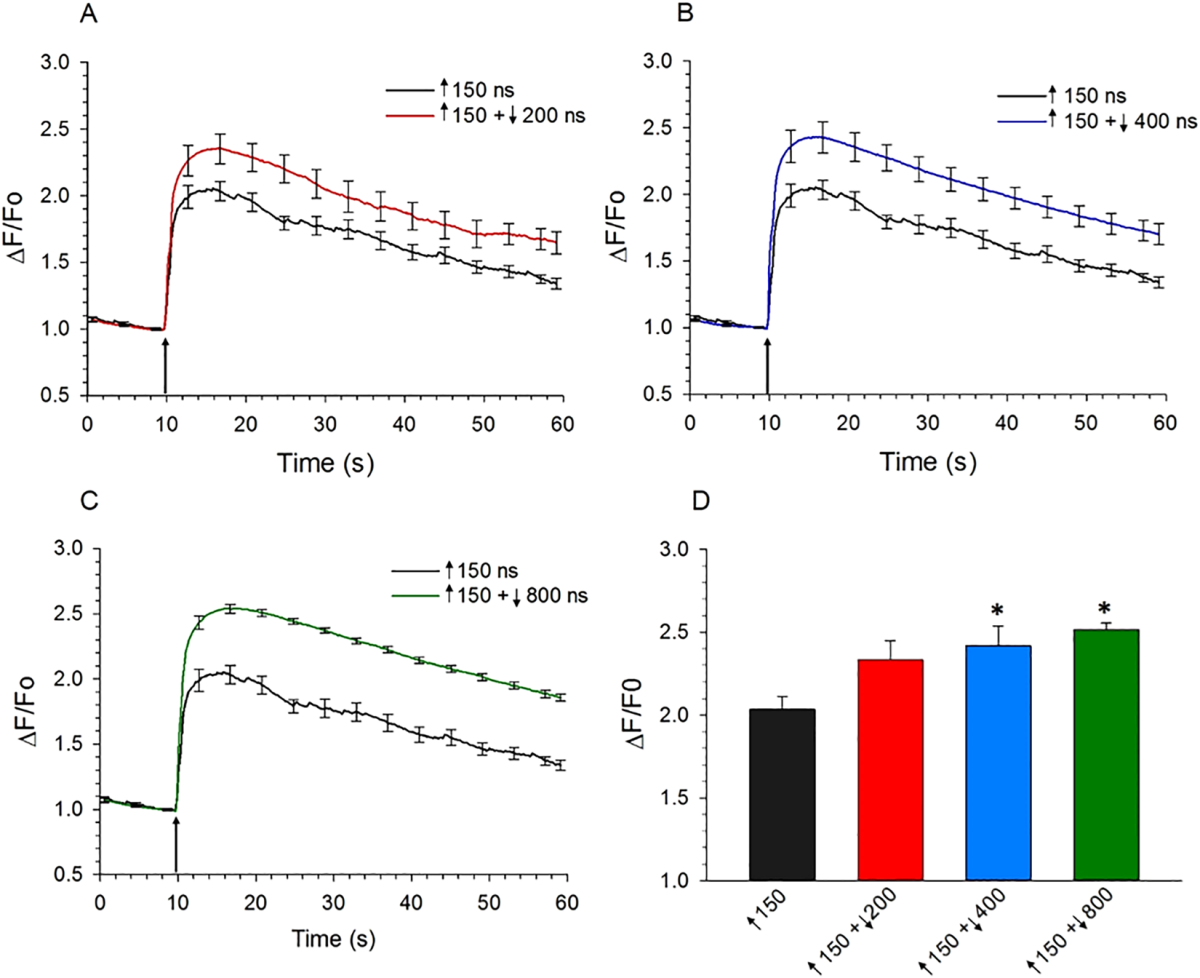
Comparison of the effect of asymmetrical bipolar pulses vs a unipolar pulse on [Ca^2+^]_i_ when the duration of the negative phase was increased. (**A**) Averaged cell responses ± s.e.m. for the rise in [Ca^2+^]_i_ in cells exposed to a 150 ns unipolar pulse (n = 8) vs a ↑150 + ↓200 ns bipolar pulse n = 8). The E-field amplitude for each phase was 3.1 kV/cm and the arrow indicates when the pulse was delivered to the cell. (**B**) Same as in (**A**) except that the bipolar pulse was ↑150 + ↓400 ns (n = 10), and in (**C**) 150 + ↓800 ns (n = 10). (**D**) Bar graph showing the mean ± s.e.m. for the maximal increase in [Ca^2+^]_i_ for the responses shown in (**A**–**C**). ^*^p < 0.05, significantly different from the 150 ns unipolar pulse.

As shown in Fig. 2, Ca^2+^ influx stimulated by single unipolar pulses increased in magnitude with pulse duration. Thus, increasing the duration of the negative phase of a bipolar pulse would presumably result in more electropermeabilization and hence more Ca^2+^ influx, resulting in even greater increases in [Ca^2+^]_i_ when compared to that evoked by a symmetrical ↑150 + ↓150 bipolar pulse. To examine this possibility, the positive pulse phase was kept constant at 150 ns and the duration of the negative pulse phase was varied from 200 up to 800 ns, both phases set at an E-field of 3.1 kV/cm. The specific asymmetrical bipolar pulses used in the analysis were ↑150 + ↓200, ↑150 + ↓400 or ↑150 + ↓800 ns (Fig. 5A). As shown in Fig. 6A–D, the magnitude of the rise in [Ca^2+^]_i_, when compared to that triggered by a 150 ns unipolar pulse, increased in concert with the increase in duration of the negative pulse phase (One-way ANOVA; p < 0.05). These results are consistent with the demonstration in CHO cells that a ↑300 + ↓900 bipolar pulse caused more YO-PRO-1 uptake than a 300 ns unipolar pulse^22^. Table 1 shows further that the Ca^2+^ responses evoked by each asymmetrical bipolar pulse were also greater than those evoked by a symmetrical ↑150 + ↓150 bipolar pulse. *Post hoc* analysis revealed that Ca^2+^ responses elicited by ↑150 + ↓200 bipolar pulses were not significantly different from those produced by 150 ns unipolar pulses (*p* > 0.05) whereas responses to bipolar pulses of ↑150 + ↓400 and ↑150 + ↓800 were significantly different from those evoked by 150 ns unipolar pulses (*p* < 0.05 and *p* < 0.005, respectively). Although the magnitude of the rise in [Ca^2+^]_i_ tended to increase in a linear fashion with the duration of the negative phase (Fig. 6D), the differences were not statistically significant within the group. These results showing that increasing the duration of the negative pulse phase did not attenuate Ca^2+^ influx but instead enhanced it prompted us to evaluate other bipolar pulse waveforms for achieving cancellation.

### Bipolar pulses with a long duration, low E-field amplitude negative pulse phase attenuated Ca^2+^ influx

**Figure 7:**
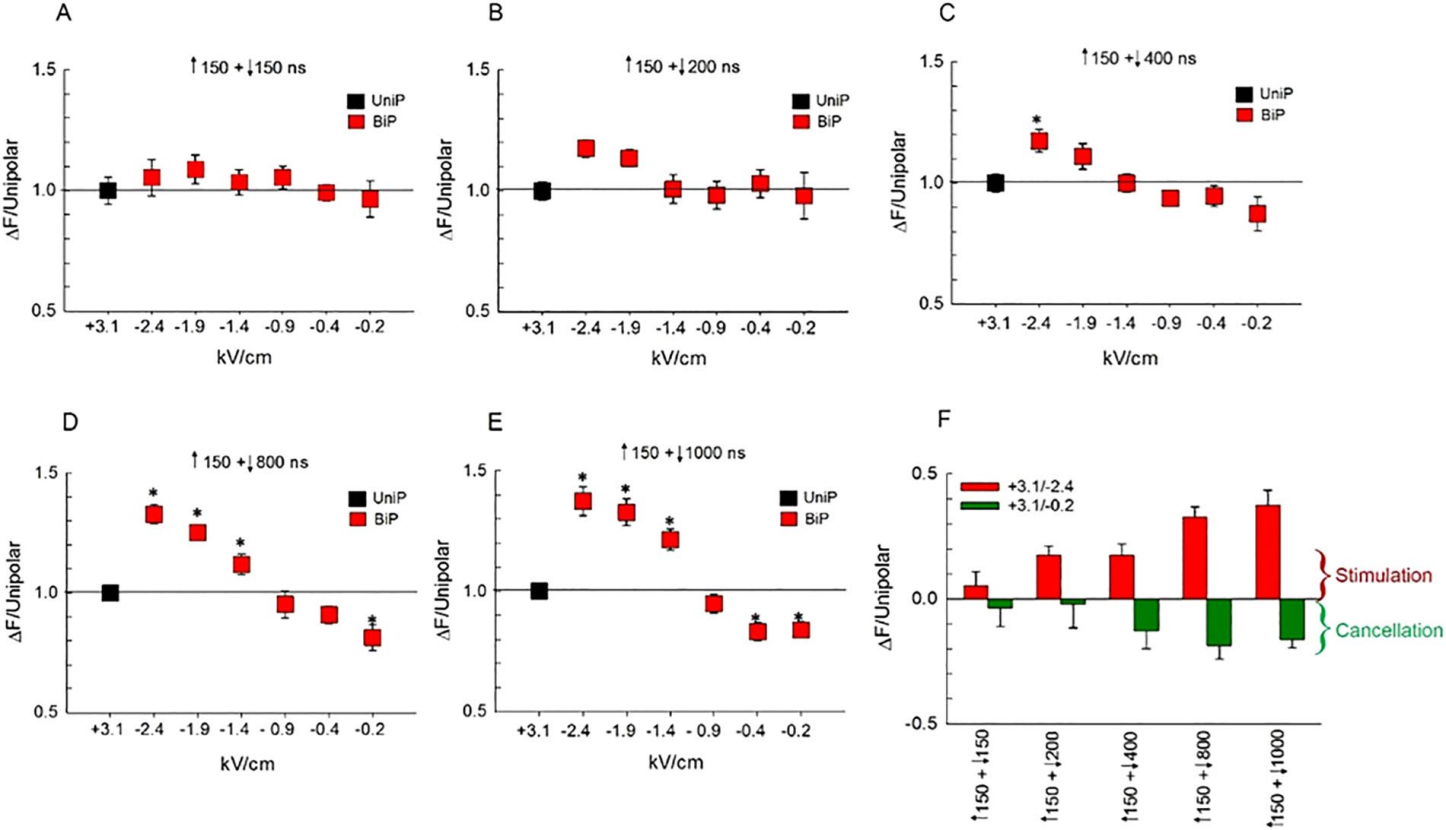
Effect of simultaneously decreasing the amplitude and increasing the duration of the negative phase of a bipolar pulse on [Ca^2+^]_i_. For panels (A–E), the amplitude of Ca^2+^ transients (mean ± s.e.m.) triggered by bipolar pulses was normalized to that elicited by a 150 ns unipolar pulse (n = 6–9) set to an E-field of 3.1 kV/cm, and plotted as a function of the E-field amplitude of the negative pulse (represented as negative values). Pulse duration combinations were as follows: (**A**) ↑150 + ↓150 ns (n = 7–9), (**B**) ↑150 + ↓200 ns (n = 5–9), (**C**) ↑150 + ↓400 ns (n = 6–11), (**D**) ↑150 + ↓800 ns (n = 10–16) and (**E**) ↑150 + ↓1000 ns (n = 7–12). (**F**) Bar graph showing the mean ± s.e.m. for the maximal increase (Stimulation) or decrease (Cancellation) in the amplitude of the Ca^2+^ transients for the responses shown in (**A**–**E**) relative to that elicited by a 150 ns unipolar pulse. The E-field of the negative phase of the pulse was either 2.4 or 0.2 kV/cm. ^*^p < 0.05, significantly different from corresponding 150 ns unipolar pulse.

The results of studies in which uptake of YO-PRO-1 served as an indicator of membrane electropermeabilization revealed that bipolar cancellation could be achieved by manipulating the parameters of the negative pulse phase in specific ways. Gianulis *et al*.^21^ observed bipolar cancellation when the amplitude of the E-field of the negative pulse phase was reduced to 35% of the initial positive pulse phase. A subsequent study from the same group reported also that optimal cancellation by a single or multiple nsPEFs in different cell types could be achieved by a negative phase E-field amplitude ~50% lower than the positive phase^25^. With respect to pulse duration, YO-PRO-1 uptake into CHO cells exposed to a 900 ns unipolar pulse was reduced 73% by a ↑900 + ↓300 bipolar pulse^22^. In view of these findings, we tested for possible cancellation of Ca^2+^ influx various bipolar pulse waveforms where, relative to the positive phase, the duration of the negative pulse phase was increased and the E-field amplitude was decreased. Essentially, for each negative pulse phase duration tested between 150 and 1000 ns, we evaluated the impact of progressively decreasing the E-field amplitude from 2.4 to 0.2 kV/cm (Fig. 7). Representative traces of each waveform are depicted in Fig. 5B. A 150 ns unipolar pulse with an E-field amplitude of 3.1 kV/cm was used in all experiments to obtain a control response to which the amplitude of the Ca^2+^ transient evoked by the asymmetrical bipolar pulse was normalized.

**Figure 8:**
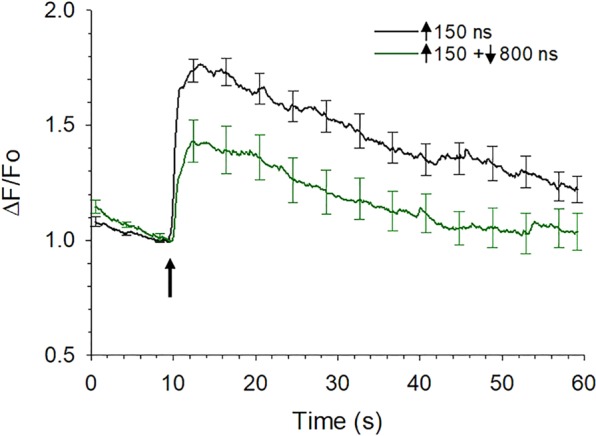
Comparison of the effect of asymmetrical bipolar pulses vs a unipolar pulse on [Ca^2+^]_i_ when the duration of the negative phase was increased and the amplitude decreased. Averaged cell responses ± s.e.m for cells exposed to a 150 ns unipolar pulse vs a ↑150 + ↓800 ns pulse (n = 15–10) in which the positive pulse phase was set to 3.1 kV/cm and negative pulse phase set at 0.2 kV/cm. The arrow indicates when the pulse was delivered to the cells.

Consistent with the experiments described in the previous section, increasing the duration of the negative pulse phase led to enhanced stimulation, especially at higher E-fields (compare for example Fig. 7A,E at 2.4 kV/cm; Fig. 7F). Interestingly, the stimulatory effect of the negative pulse progressively subsided by decreasing the E-field amplitude from 2.4 to 0.2 kV/cm for all pulse durations tested (Fig. 7A thru E; summarized in Fig. 7F). In fact, significant cancellation was observed for very long durations and low E-field amplitudes, in particular with asymmetrical bipolar pulses of ↑150 + ↓800 ns at an E-field of 0.2 kV/cm (Fig. 7D; *p* < 0.01) and ↑150 + ↓1000 ns at an E-field ≤ 0.4 kV/cm (Fig. 7E; *p* < 0.01). Figure 8 shows the response of the cells to a ↑150 + ↓800 ns pulse, negative phase set to 0.2 kV/cm, where it can be seen that compared with the Ca^2+^ response elicited by the 150 ns unipolar pulse, not only was the magnitude of the rise in [Ca^2+^]_i_ decreased but also [Ca^2+^]_i_ returned to pre-stimulus levels during the 50-s post-pulse monitoring period. Indeed, the Ca^2+^ response triggered by these bipolar pulse waveforms now more closely resembled that evoked by the physiologic stimulus, activation of nicotinic cholinergic receptors^26^. Due to the technical limitations of our bipolar pulse exposure system, we could not determine if maximal cancellation of Ca^2+^ influx was achieved since this would require negative pulse phases of even lower E-field amplitude and/or longer duration, which are not possible with our setup.

Table 1 provides a summary of mean data for % stimulation or cancellation of Ca^2+^ influx by the various pulse parameter combinations. Cancellation reached a maximum of 43% when the positive phase was 150 ns at 3.1 kV/cm, and the negative phase was 800 ns at 0.2 kV/cm. We currently have no plausible explanation for why a bipolar pulse consisting of a prolonged duration, low amplitude negative phase was needed to cause cancellation of Ca^2+^ influx in these cells. Our data are nevertheless in general agreement with the observation that reducing the E-field amplitude of the negative relative to the initial positive pulse phase has a cancellation effect^21,25^.

### Stimulation of Ca^2+^ influx by symmetrical bipolar pulses was converted to cancellation when VGCCs were blocked

As indicated previously, strong bipolar cancellation of Ca^2+^ influx has been reported for symmetrical bipolar pulses in CHO cells that lack VGCCs^20^. In contrast, in chromaffin cells that express multiple types of VGCCs^1^, bipolar cancellation by symmetrical bipolar pulses was not achieved (Fig. 2) even though our recent report showed that ~30–40% of the rise in [Ca^2+^]_i_ triggered by 150 ns unipolar pulses occurred through a process of electronanoporation^18^. One potential factor that could explain the differential response of chromaffin cells relative to CHO cells may be attributed to the presence of VGCCs in the former but not in the latter. Since VGCCs respond in an all-or-none fashion to 5 ns unipolar pulses^26^ and 60–70% of Ca^2+^ influx triggered by longer (≥150 ns) unipolar pulses is passing through VGCCs^18^, it is possible that bipolar pulses are unable or are less efficient at cancelling the Ca^2+^ influx component involving VGCCs, or that the Ca^2+^ that enters the cells via VGCCs affects how the cell responds to a bipolar pulse.

To explore these possibilities, we determined whether conducting experiments under conditions that would eliminate the contribution of VGCCs to nsPEF-evoked responses would show cancellation of Ca^2+^ influx by symmetrical bipolar pulses, as shown for cells that do not express VGCCs. For this determination, chromaffin cells were pre-treated with a combination (cocktail) of Ca^2+^ channel blockers and exposed to a ↑150 + ↓150 ns bipolar pulse. The cocktail of Ca^2+^ channel blockers comprised 100 nM ω-agatoxin GVIA (P/Q-type) + 20 nM ω-conotoxin (N-type) + 20 µM nitrendipine (L-type), which we previously determined was effective for blocking Ca^2+^ influx mediated by VGCCs in cells exposed to the pulse durations used in this study^18^. In other experiments, cells were pre-treated with dihydropyridines alone at high concentrations (20 µM nitrendipine, 20 µM nimodipine or 40 µM nimodipine), which we previously also determined caused substantial inhibition of Ca^2+^ influx triggered by ≥150 ns pulses^18^. High concentrations of dihydropyridines ensured that both Ca_V_1.2 and Ca_V_1.3 isoforms of L-type channels, which are both expressed in bovine chromaffin cells^27^, were effectively inhibited since Ca_V_1.3 channels are less sensitive to dihydropyridines than Ca_V_1.2^28^. That this treatment significantly inhibited Ca^2+^ influx^10^ most likely meant that the other types of VGCCs present in these cells were partially blocked^29^.

**Figure 9:**
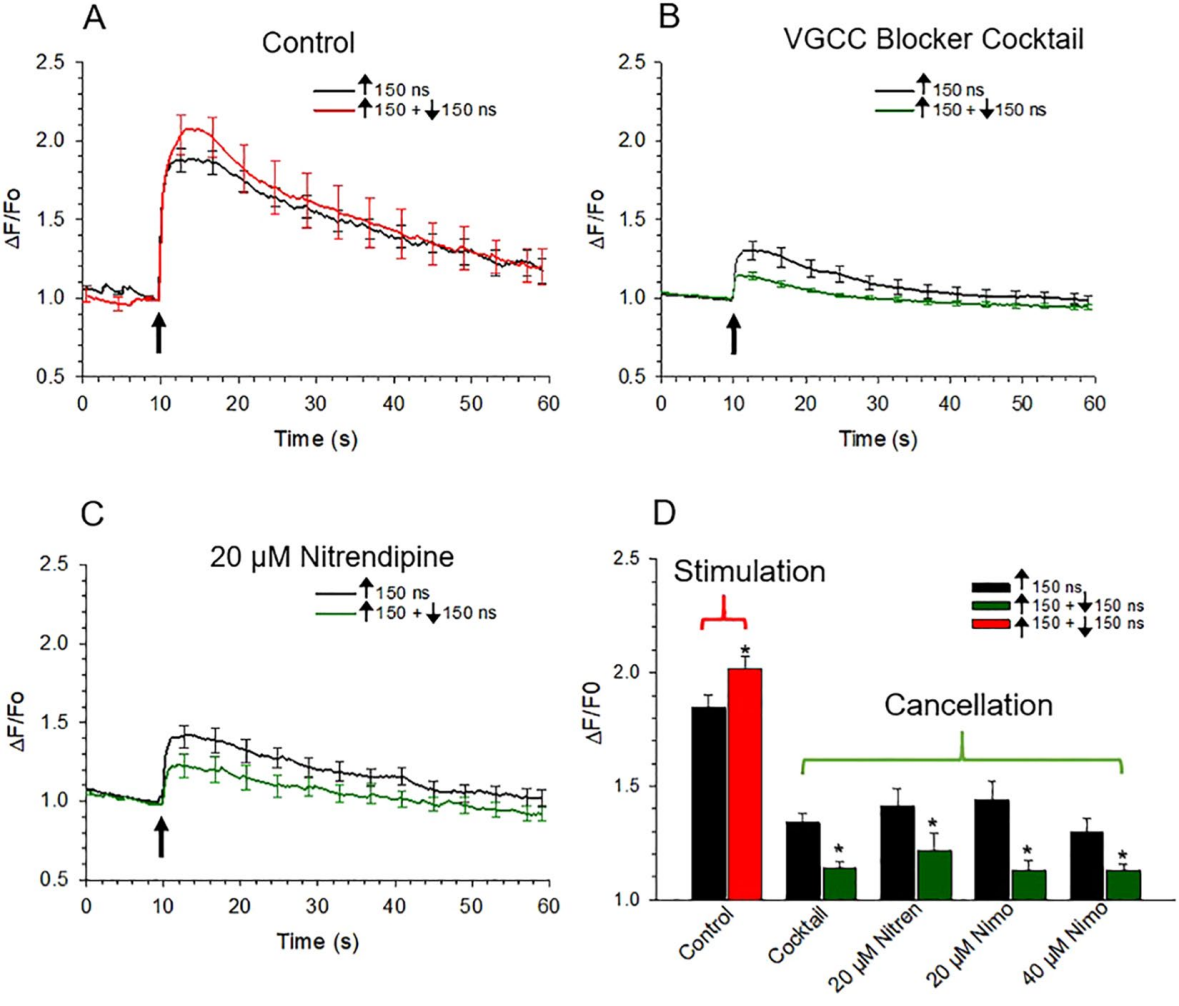
Effect of blocking VGCCs on Ca^2+^ responses elicited by symmetrical bipolar pulses. Averaged cell responses ± s.e.m for cells exposed to a 150 ns unipolar pulse vs a ↑150 + ↓150 ns symmetrical bipolar pulse with all pulse phases set to 3.1 kV/cm. Ca^2+^ responses were monitored in cells in the absence of VGCC blockers (**A**) Control (n = 8–6), or in the presence of a (**B**) cocktail of VGCC blockers containing 100 nM ω-agatoxin, 20 nM ω-conotoxin and 20 µM nitrendipine (n = 18–25) or (**C**) 20 µM nitrendipine (n = 32–34). The arrow indicates when the pulse was delivered to the cells. (**D**) Bar graph showing the mean ± s.e.m. for the maximal increase in [Ca^2+^]_i_ elicited by a 150 ns unipolar pulse vs a ↑150 + ↓150 ns symmetrical bipolar pulse in the absence or presence of the VGCC blocker cocktail, 20 µM nitrendipine (Nitren) or 20 μM (n = 13–10) or 40 μM (n = 9–9) nimodipine (Nimo). Cells were pre-treated with the blockers for 1 hr at room temperature. ^*^p < 0.05, significantly different from the corresponding unipolar pulse.

Similar to the results shown in Fig. 2, those in Fig. 9A show that a symmetrical bipolar ↑150 + ↓150 ns pulse evoked a rise in [Ca^2+^]_i_ that was larger by around 18% than that elicited by the corresponding 150 ns unipolar pulse. When cells were treated with the cocktail of VGCC blockers, Ca^2+^ influx elicited by a 150 ns pulse was inhibited by around 66% (Fig. 8A,B), which is in agreement with our previous findings^18^. We attributed the remaining 34% of Ca^2+^ influx to electropermeabilization. Figure 8A,B also show that the presence of the Ca^2+^ channel blockers converted symmetrical bipolar pulse-induced stimulation to cancellation. That is, when VGCCs were blocked, Ca^2+^ influx was reduced 50% by the symmetrical bipolar pulse. Figure 9C shows further that bipolar pulse-induced Ca^2+^ influx in the presence of 20 µM nitrendipine was likewise attenuated to a comparable degree. Results similar to those shown in Fig. 9C were also found for 20 µM nimodipine and 40 µM nimodipine (not shown). Figure 9D summarizes the results where we observed 60%, 49%, 70% and 59% attenuation of the bipolar-mediated Ca^2+^ response with a cocktail of VGCC inhibitors, 20 µM nitrendipine, 20 µM nimodipine, and 40 µM nimodipine, respectively. In each case, the difference between the corresponding unipolar pulse control (i.e., in the presence of the VGCC blocker) reached statistical significance (*p* < 0.01 for all treatments). These results are evidence that bipolar pulses were cancelling the portion of Ca^2+^ influx attributable to Ca^2+^ permeation mediated by electronanoporation and not Ca^2+^ influx via VGCCs. They also beg the question of why Ca^2+^ influx due to electronanoporation was now achievable when Ca^2+^ influx via VGCCs was suppressed. The answer to this question awaits future investigation^30^.

The levels of cancellation achieved by suppressing VGCCs were slightly greater than those achieved by asymmetrical bipolar pulses producing significant and optimal cancellation (Table 1). This difference could be because full cancellation of Ca^2+^ influx was not achievable with the range of asymmetrical bipolar pulse waveforms available with our current exposure setup. It could also be because any depolarizing stimulus that causes Ca^2+^ entry via VGCCs in chromaffin cells, such as nicotinic receptor stimulation as well as nsPEF, may also trigger some CICR due to activation of ryanodine receptors^30–33^. Thus, blocking Ca^2+^ influx via VGCCs would also prevent CICR, causing an apparent enhanced cancellation effect relative to cells exposed to asymmetric bipolar pulses with VGCC responses intact. We did not test for the presence of CICR since it is a response that is secondary to Ca^2+^ influx via VGCCs and thus outside the scope of the present study, which was mainly focused on determining pulse parameters and conditions that would allow us to detect bipolar cancellation of Ca^2+^ influx.

### Blocking VGCCs enhanced the cancellation of Ca^2+^ influx by asymmetrical bipolar pulses

**Figure 10:**
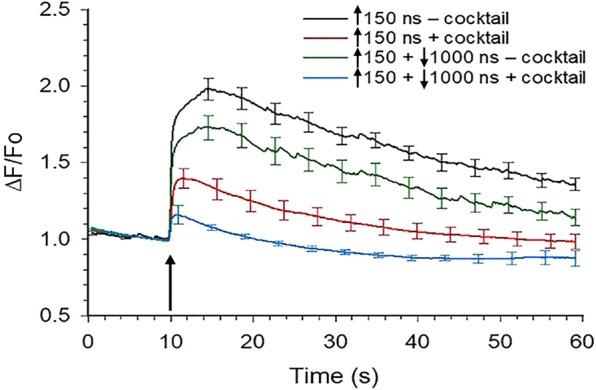
Effect of blocking VGCCs on Ca^2+^ responses elicited by asymmetrical bipolar pulses. Averaged cell responses ± s.e.m. for the rise in [Ca^2+^]_i_ in cells exposed to a 150 ns unipolar pulse vs a ↑150 + ↓1000 ns bipolar pulse in the presence of a cocktail of VGCC blockers (+cocktail; n = 7–12), whose composition was identical to that described in Fig. 8, or in the absence of the cocktail (−cocktail; n = 7–9). Cells were pre-treated with the blockers for one hr at room temperature. Positive and negative pulse phases of the pulse were set to 3.1 and 0.4 kV/cm, respectively, and the arrow indicates when the pulse was applied to the cells. ^*^p < 0.05, significantly different from the response triggered by the corresponding 150 ns unipolar pulse. The arrow indicates when the pulse was delivered to the cells.

Since cancellation of Ca^2+^ influx by symmetrical bipolar pulses was observed only when VGCCs were blocked, we explored how Ca^2+^ influx via VGCCs impacted the level of cancellation produced by an asymmetrical bipolar pulse having a long duration (1000 ns), low E-field amplitude (0.4 kV/cm) negative pulse phase. For these experiments, cells were exposed to a 150 ns unipolar pulse or to a ↑150 + ↓1000 ns bipolar pulse in the presence and absence of the VGCC blocker cocktail. As shown in Fig. 10 and Table 1, the level of cancellation of Ca^2+^ influx by the asymmetrical bipolar pulse was significantly greater when VGCCs were blocked (36% vs 58% in the absence and presence of the VGCC blocker cocktail, respectively). These results again demonstrate that in chromaffin cells, bipolar pulses appear to be cancelling the portion of Ca^2+^ influx attributable to Ca^2+^ permeation mediated by electronanoporation. They also provide additional evidence that Ca^2+^ influx via VGCCs may impact cancellation efficiency.

### Summary

This study provides new insight into the potential application of bipolar pulses for modulating nsPEF-evoked Ca^2+^ responses in excitable cells. Using neuroendocrine chromaffin cells isolated from the adrenal medulla as a cell model, we had previously reported^10^ and show here also that exposing these cells to unipolar pulses that are ≥150 ns in duration elicit an increase in [Ca^2+^]_i_ that is mediated by Ca^2+^ influx via both VGCCs and putative plasma membrane nanopores, i.e. electropores. We now show that depending on the shape of the pulse waveform, exposing chromaffin cells instead to a bipolar pulse can partially cancel the portion of Ca^2+^ influx attributed to electropores, thereby attenuating an undesirable pathway of Ca^2+^ entry into the cells while leaving all-or-none VGCC activation intact. This finding, together with reports from other groups showing that bipolar pulses can cause less cell death of CHO, U937 and Jurkat cells than unipolar pulses^24^, provides incentive for continuing studies that explore the range of nsPEF parameters that can be used to drive neuromodulation safely and potentially non-invasively in the future.

Regarding mechanisms, bipolar stimulation of Ca^2+^ influx, where the second phase of the bipolar pulse acts like a 2^nd^ stimulus, most likely reflects an increase in the number of electropores. However, mechanisms underlying bipolar cancellation are not yet well defined and several have been proposed to explain the phenomenon. They include: (1) assisted membrane discharge^20^; (2) electropermeabilization of membranes as a two-step chemical process^20^; (3) reverse electrophoretic ion transfer^20,34^; (4) localized membrane charging and discharging^22^ and (5) nanopore occlusion^35^. Adding to the complexity of the bipolar cancellation phenomenon in chromaffin cells is the demonstration that for symmetrical bipolar pulses, Ca^2+^ influx via VGCCs can determine whether there is cancellation or stimulation. Future experiments will be carried out to test these various hypotheses.

In conclusion, the demonstration in this study that the shape of bipolar nsPEFs could be altered in manner to allow for titrating the response of chromaffin cells may pave the way in the future for remote and non-invasive control of these and other types of excitable cells *in vivo*.

## Materials and Methods

### Chromaffin cell culturing and preparation

Fresh bovine adrenal glands were obtained from a local slaughterhouse (Wolf Pack Meats, University of Nevada, Reno). After the outer adrenal cortex was removed by dissection, chromaffin cells were isolated by collagenase digestion of the adrenal medulla and maintained in suspension culture in Ham’s F-12 medium supplemented with 10% bovine calf serum, 100 U/ml penicillin, 100 µg/ml streptomycin, 0.25 µg/ml fungizone, and 6 µg/ml cytosine arabinoside at 36.5 °C under a humidified atmosphere of 5% CO_2_ as previously described^36^. Cells were used up until 2 weeks in culture. For nsPEF exposure, the large aggregates of cells that form in suspension culture were dissociated into single isolated cells with the protease dispase^37^ and plated onto fibronectin-coated 35 mm glass bottom dishes. Once attached, cells retained their spherical morphology and were used for a period not exceeding two days after attachment.

### Fluorescence imaging of intracellular Ca^2+^ levels

Changes in [Ca^2+^]_i_ were monitored in cells labeled with the Ca^2+^-sensitive fluorescent indicator, Calcium Green-1(Ex_480 nm_ and Em_535 nm_) as previously described^18^. For dye loading, the cells were incubated with 1 µM Calcium Green-1 for 45 min at 37 °C in a balanced salt solution (BSS) containing 0.1% bovine serum albumin (BSA) and (in mM): 145 NaCl, 5 KCl, 1.2 NaH_2_PO_4_, 2 CaCl_2_, 1.3 MgCl_2_, 10 glucose, and 15 HEPES, pH 7.4. For fluorescence monitoring, cells were washed twice with dye-free BSS lacking BSA and placed on the stage of a Nikon TE2000 epifluorescence microscope equipped with a 100X objective. For Ca^2+^ free experiments cells were bathed in Ca^2+^-free BSS Ca^2+^ containing 1 mM EGTA. Fluorescence images of the cells were captured before, during and after application of a stimulus by an iXonEM + DU-897 EMCCD camera (Andor Technology Ltd., Belfast, UK) using the open source microscopy software Micro-Manager (version 1.4, Vale Lab, UCSF, San Francisco, CA). The exposure time of the camera was set to 100 ms and images were captured at a rate of 7.5 Hz. Bright field images were obtained at the start and end of an experiment, and all experiments were carried out at ambient room temperature.

### nsPEF exposure

The custom-designed high-voltage biphasic nanosecond pulse generator used to expose chromaffin cells to nsPEF has been previously described^18^ and complete fabrication details are given in Ryan *et al*.^38^. The unit has two independent circuits that deliver positive and negative pulses ranging from 150 to 1000 ns, with the ability to adjust the pulse widths and amplitudes of the positive and negative pulses separately. For cell exposure, single unipolar and bipolar pulses were delivered to an attached cell via a pair of cylindrical tungsten rod electrodes (127 µm diameter) with their tips spaced 100 µm apart. The cell to be investigated was positioned at the center of the gap between the electrode tips, which were positioned 40 µm above the bottom of the dish using a motorized micromanipulator (model MP-225, Sutter Instruments, Novata, CA). A LabVIEW program (version 8.01) was used to control the delivery of pulses. Each cell was exposed to a single unipolar or bipolar pulse only once. As reported in Bagalkot *et al*.^18^, the E-field distribution in the vicinity and at the location of the target cell was computed using the commercially available Finite-Difference Time-Domain (FDTD) software package SEMCAD X (version 14.8.5, SPEAG, Zurich, Switzerland). The simulations showed that the E-field at the location of the cell was essentially uniform and that minor differences in electrode placement among cells would have negligible impact on the E-field amplitude to which a cell was exposed^18^.

### Image processing and data analysis

Fluorescence images for each cell were continuously recorded during a period spanning 10 s prior to nsPEF exposure to obtain baseline, and then for a period spanning 50 s after nsPEF exposure. Images were analyzed using the public domain image processing software ImageJ (https://imagej.nih.gov/ij/). Changes in cell fluorescence intensity (ΔF) were calculated by subtracting background fluorescence from the fluorescence of the cell (ΔF = F_cell_ − F_background_). The values were then normalized to the fluorescence intensity value at the time when the pulse was applied (F_0_). All experiments were replicated using cells from different cell preparations and different days in culture. Results were expressed as the mean ± standard error of the mean (s.e.m.). Statistical analyses, which were performed using SigmaPlot 13 software (Systat software, Inc., San Jose, CA), consisted of an unpaired two-tailed t-test when the means of two groups were compared, or a one-way ANOVA test followed by Tukey *post hoc* multiple range tests for multiple group comparisons. *p* < 0.05 was considered statistically significant.
